# Listening panel agreement and characteristics of lung sounds digitally recorded from children aged 1–59 months enrolled in the Pneumonia Etiology Research for Child Health (PERCH) case–control study

**DOI:** 10.1136/bmjresp-2017-000193

**Published:** 2017-06-30

**Authors:** Eric D McCollum, Daniel E Park, Nora L Watson, W Chris Buck, Charatdao Bunthi, Akash Devendra, Bernard E Ebruke, Mounya Elhilali, Dimitra Emmanouilidou, Anthony J Garcia-Prats, Leah Githinji, Lokman Hossain, Shabir A Madhi, David P Moore, Justin Mulindwa, Dan Olson, Juliet O Awori, Warunee P Vandepitte, Charl Verwey, James E West, Maria D Knoll, Katherine L O'Brien, Daniel R Feikin, Laura L Hammit

**Affiliations:** 1 Eudowood Division of Pediatric Respiratory Sciences, Johns Hopkins School of Medicine, Baltimore, Maryland, USA; 2 Department of International Health, Johns Hopkins Bloomberg School of Public Health, Dhaka, Bangladesh; 3 Department of International Health, International Vaccine Access Center, Johns Hopkins Bloomberg School of Public Health, Baltimore, Maryland, USA; 4 The Emmes Corporation, Rockville, Maryland, USA; 5 Department of Pediatrics, University of California Los Angeles, Maputo, Mozambique; 6 International Emerging Infections Program, Global Disease Detection Center, Thailand Ministry of Public Health – US Centers for Disease Control and Prevention Collaboration, Nonthaburi, Thailand; 7 National Health Service Highland, Inverness, UK; 8 The Medical Research Council, Basse, The Gambia; 9 Department of Electrical and Computer Engineering, Johns Hopkins University, Baltimore, USA; 10 Department of Paediatrics and Child Health, Stellenbosch University, Tygerberg, South Africa; 11 Division of Paediatric Pulmonology, University of Cape Town, Cape Town, South Africa; 12 Respiratory Vaccines, Center for Vaccine Sciences, icddr,b, Dhaka, Bangladesh; 13 Medical Research Council, Respiratory and Meningeal Pathogens Research Unit, University of the Witwatersrand, Johannesburg, South Africa; 14 Department of Science and Technology/National Research Foundation, South African Research Chair: Vaccine Preventable Diseases, University of the Witwatersrand, Johannesburg, South Africa; 15 Department of Paediatrics, University of the Witwatersrand, Chris Hani Baragwanath Academic Hospital, Johannesburg, South Africa; 16 Department of Paediatrics and Child Health, University Teaching Hospital, Lusaka, Zambia; 17 Department of Pediatrics, Section of Infectious Disease, Center for Global Health, University of Colorado, Colorado, USA; 18 Kenya Medical Research Institute Wellcome Trust Research Programme, Kilifi, Kenya; 19 Queen Sirikit National Institute of Child Health, Rangsit University, Bangkok, Thailand; 20 Division of Viral Diseases, Centers for Disease Control and Prevention, Atlanta, Georgia, USA

**Keywords:** pneumonia, paediatric lung disaese, respiratory infection

## Abstract

**Introduction:**

Paediatric lung sound recordings can be systematically assessed, but methodological feasibility and validity is unknown, especially from developing countries. We examined the performance of acoustically interpreting recorded paediatric lung sounds and compared sound characteristics between cases and controls.

**Methods:**

Pneumonia Etiology Research for Child Health staff in six African and Asian sites recorded lung sounds with a digital stethoscope in cases and controls. Cases aged 1–59 months had WHO severe or very severe pneumonia; age-matched community controls did not. A listening panel assigned examination results of normal, crackle, wheeze, crackle and wheeze or uninterpretable, with adjudication of discordant interpretations. Classifications were recategorised into any crackle, any wheeze or abnormal (any crackle or wheeze) and primary listener agreement (first two listeners) was analysed among interpretable examinations using the prevalence-adjusted, bias-adjusted kappa (PABAK). We examined predictors of disagreement with logistic regression and compared case and control lung sounds with descriptive statistics.

**Results:**

Primary listeners considered 89.5% of 792 case and 92.4% of 301 control recordings interpretable. Among interpretable recordings, listeners agreed on the presence or absence of any abnormality in 74.9% (PABAK 0.50) of cases and 69.8% (PABAK 0.40) of controls, presence/absence of crackles in 70.6% (PABAK 0.41) of cases and 82.4% (PABAK 0.65) of controls and presence/absence of wheeze in 72.6% (PABAK 0.45) of cases and 73.8% (PABAK 0.48) of controls. Controls, tachypnoea, >3 uninterpretable chest positions, crying, upper airway noises and study site predicted listener disagreement. Among all interpretable examinations, 38.0% of cases and 84.9% of controls were normal (p<0.0001); wheezing was the most common sound (49.9%) in cases.

**Conclusions:**

Listening panel and case–control data suggests our methodology is feasible, likely valid and that small airway inflammation is common in WHO pneumonia. Digital auscultation may be an important future pneumonia diagnostic in developing countries.

## Introduction

Paediatric pneumonia is a major cause of global mortality.^1^ The WHO case management algorithm for childhood pneumonia was developed to be practical and diagnostically sensitive, so frontline health workers in low-resource settings could empirically treat possible bacterial pneumonia using basic skills.^2^ While the algorithm’s high sensitivity has increased antibiotic use in children previously untreated for bacterial pneumonia,^3^ it comes at the expense of misdiagnosis and likely antibiotic overuse.^4–7^

During pneumonia, a complex inflammatory cascade causes the lung’s gas exchange units, alveoli, to collapse.^8^ When a child with pneumonia inhales, alveoli can explosively reopen, causing popping sounds called crackles.^9^ When crackles are present, the likelihood of pneumonia increases.^10 11^ Although a traditional stethoscope is inexpensive and can be used to identify crackles, interpretations are plagued by subjectivity between listeners evaluating the same lung sound and also from breath-to-breath inspiratory and expiratory variations in children that can change the character of lung sounds during and between examinations.^12 13^ Presumably due to these factors, and the challenge of teaching auscultation, the WHO did not incorporate auscultation as a diagnostic into its pneumonia management algorithm for frontline practitioners.^2^

Technological advances may help to overcome interpretation inconsistencies by producing high-quality, permanent lung recordings that can be systematically interpreted by humans or computers.^14 15^ Modern digital stethoscope designs allow sounds to be transduced with high fidelity and recorded and saved as an audio file.^16^ Amplification and filtering techniques can optimise sound quality for acoustic human interpretation and, if computerised acoustic analysis techniques are also applied, visual interpretation.^17^ Moreover, mathematical methods can now deconstruct sound data into quantitative patterns for computer analysis, bypassing human interpretation altogether.^18^ Automated interpretation of lung sounds with a handheld device could be especially powerful in low-resource settings that lack trained paediatric healthcare providers. Digital auscultation research to date has largely focused on adults in high-income settings^18^ and that research is not likely relevant to the application of digital auscultation to children in low-income countries. If digital auscultation is to have a role in low-resource countries, relevant paediatric research is needed.

We recorded lung sounds with digital stethoscopes from a subset of children aged 1–59 months in six African and Asian sites in the Pneumonia Etiology Research for Child Health (PERCH) study,^19^ aiming to determine the feasibility of recording quality lung sounds from children in noisy settings, to develop and assess a method for adjudicating lung sound examinations acoustically interpreted by humans, identify predictors of listener disagreement to inform future research methodology in developing countries and, lastly, describe and compare digitally recorded lung sound characteristics among cases and controls in PERCH.

## Patients and methods

### PERCH enrolment

PERCH was a 2 year case–control study of severe childhood pneumonia aetiology in seven countries in Africa and Asia.^19^ Eligible cases were hospitalised children aged 1–59 months with WHO-defined severe or very severe pneumonia (panel).^20^ Wheezing cases whose chest indrawing resolved after bronchodilators were excluded.^20^ Randomly selected, age-matched children were enrolled as community controls if they did not meet the case definition, even if they had respiratory symptoms (panel).^21^ Staff were trained on clinical measurements and specimen collection (table 1).^20^

**Table 1:** Panel

Study definitions			
Cases			
WHO severe pneumonia	Cough and/or difficult breathing with lower chest indrawing and no WHO danger signs		
WHO very severe pneumonia	Cough and/or difficult breathing with at least one danger sign (central cyanosis, difficulty breastfeeding or drinking, vomiting everything, convulsions, lethargy, or unconsciousness, head nodding)		
Controls			
ARI control	Cough or runny nose reported or if (A) ear discharge, wheeze or difficulty breathing *and* (B) either fever (temperature >38.0°C or reported fever in the past 48 hours) or sore throat were reported.		
Non-ARI control	Does not meet definition of case or ARI control.		
Lung sounds	Description	Inspiration	Expiration
Normal	Soft, not musical	Throughout	Early only
Crackle	Short, explosive, not musical, popping; usually repetitive	Primarily (but can be variable)	Less common and usually with inspiratory crackles
Wheeze	Musical, long duration; can be high or low pitch	Possible	Primarily, prolonged
Uninterpretable	Persistent crying or poor quality such that no full breath sounds are heard	Yes	Yes
Upper airway noises, not stridor	Generally louder at cheek, may mimic a low pitch wheeze or have ‘snorting’ quality similar to a crackle, can also be a vocalisation	Possible	Possible
Upper airway noises, primarily stridor	Generally louder at cheek, may mimic high pitched wheeze although is typically inspiratory only	Primarily	Possible, but less common

ARI, acute respiratory illness.

### Digital auscultation Enrolment

This substudy was prospectively conducted during a 14-month period (December 2012–January 2014), at six sites in a subset of PERCH cases and community controls; sampling varied by site, as described below.

### Bangladesh

Study physicians enrolled all PERCH cases in Dhaka and Matlab and about five controls per month from September to December 2013.^19^

### The Gambia

Study physicians enrolled a subset of PERCH cases and controls in Basse, time permitting, from December 2012 to October 2013.^19^

### Kenya

Nurses and clinical officers enrolled all cases, time permitting and controls when digital auscultation-trained staff conducted field visits in Kilifi from December 2012 to November 2013.^19^

### South Africa

Nurses enrolled cases not requiring mechanical ventilation, except on weekends, and controls were enrolled non-systematically according to staff workload in Soweto between December 2012 and August 2013.^19^

### Thailand

Nurses enrolled all cases and controls in Sa Kaeo and Nakhon Phanom between March 2013 and January 2014.^19^

### Zambia

A physician or clinical officer enrolled all cases and the first five controls per month in Lusaka between November 2012 and October 2013.^19^

### Sound recording procedure

**Figure 1:**
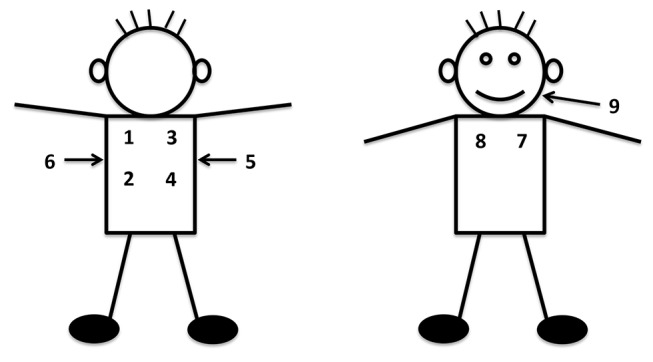
Location and sequence of listening positions for digitally recorded lung sounds.

We trained staff at the African sites to record lung sounds with a digital stethoscope (ThinkLabs ds32a®) from May to June 2012, followed by pilot data collection through October 2012. Thai and Bangladeshi staff were trained in January and June of 2013, and piloted procedures for 1 month each. Trainings were 1 day and introduced the equipment, recording and uploading procedures, troubleshooting and supervised practice. Staff recorded one lung examination per child that included lung sounds from nine sequential locations across each child’s back (four), axilla (two), chest (two) and cheek, corresponding to all lung lobes and the upper airway (figure 1). Sounds were recorded for >7 s to capture at least two respiratory cycles per location and to limit the entire procedure to about 1 min. An external microphone affixed to the stethoscope recorded ambient noise. Recordings were deidentified and then uploaded from the sound recorder to study servers. Unwanted ambient noises were removed using a novel automated multiband denoising filter developed and validated by Johns Hopkins University sound engineers (ME, DE and JEW) and physicians.^22^

### Expert listening panel and lung sound definitions

A listening panel of six paediatricians (WCB, TGP, LG, DO, WPV and CV) and two pediatric-experienced physicians (AD and JM), all highly practiced caring for African and/or Asian children with pneumonia, convened in June 2014 to formulate and refine consensus lung sound definitions for acoustic interpretation (panel). The listening panel consolidated the sound definitions in the American Thoracic Society guidelines in order to make them more pragmatic and clinically applicable for children in low-resource settings with WHO pneumonia.^17 23^ In July 2014, the panel was standardised to interpret lung sounds using a library of >100 reference digital lung sounds. Reference recordings were from children aged 1–59 months at Johns Hopkins Hospital in Baltimore, USA, collected immediately after a paediatric pulmonologist (EDM) confirmed the lung examination with a traditional stethoscope, and processed with the same denoising software used for cases and controls in PERCH.

### Expert listening panel lung examination results and adjudication process

After denoising, patient digital lung sound examinations were randomly assigned to two panellists (ie, the primary listeners) for acoustic interpretation with *Audacity* software. Listeners were masked to one another and patient information including case and control status. The cheek position was used to assess whether chest sounds were contaminated with vocalisations or upper airway noises like nasal secretions or stridor. For each patient, all eight chest position interpretations constituted one lung examination result: normal, crackle, wheeze, crackle and wheeze or uninterpretable. For example, if any chest position had a crackle, wheeze or both, then the overall lung examination result included that abnormal designation, even if other positions were normal or uninterpretable. A single chest position was uninterpretable if no full breath sounds could be distinguished. Overall, lung examination results were uninterpretable if none of the eight chest positions were interpretable by the listener. If the two primary listeners disagreed on the lung examination result, then a third panellist blinded to previous assessments was randomly selected to independently interpret the lung examination. If the third listener’s lung examination result agreed with either of the primary listeners, then the third listener’s interpretation was considered final. If not, then one panellist (DO) and a paediatric pulmonologist (EDM) decided the final result by consensus. Five per cent of case lung examinations were randomly reassigned to the same primary listener to estimate intralistener agreement at least 3 months after the initial interpretation.

Institutional review boards at the Johns Hopkins School of Public Health and all local study sites approved this research. All participants provided written informed consent.

### Statistical analysis

We evaluated agreement between and within primary listeners after grouping final panel results into dichotomous categories positive or negative for a specific lung sound (ie, any crackle, any wheeze or abnormal (any crackle or wheeze)) and including all lung examinations classified by both primary listeners as interpretable. Agreement was measured using Cohen’s kappa statistic and a kappa statistic adjusted for prevalence and listener bias (prevalence-adjusted, bias-adjusted kappa (PABAK)).^24^ Agreement strength was interpreted by the scale: ≤0, poor; 0.01–0.19, slight; 0.20–0.39, fair; 0.40–0.59, moderate; 0.60–0.79, substantial; and 0.80–1.0, perfect.^24^ To characterise predictors of between-listener disagreement, we used logistic regression models to evaluate associations of demographic and sound file characteristics with primary listener disagreement. Full models retained characteristics associated with disagreement in unadjusted models at the significance level of 0.20. Case and control lung sound recordings were compared using descriptive statistics.

## Results

A total of 1093 patients (792 cases and 301 controls) had their lung examinations recorded, denoised and evaluated by the listening panel. To evaluate the panel’s overall performance when acoustically interpreting digitally recorded lung examinations, we assessed agreement at the primary listener level for cases. For this, we excluded 83 cases with lung examinations considered uninterpretable by either primary listener, even if that examination was later arbitrated to be interpretable by the panel, leaving 709 cases (table 2) We found primary listener agreement, beyond that expected by chance, to be moderate for examinations with or without either crackle and/or wheeze (PABAK 0.50), with or without any crackle (PABAK 0.41) and with or without any wheeze (PABAK 0.45). We also evaluated the interlistener agreement by individual primary listener (table 2). Estimated agreement between the eight primary expert listeners ranged from fair to substantial; PABAKs 0.40–0.65 for examinations with or without either crackle and/or wheeze, 0.25–0.55 for examinations with or without crackle and 0.31–0.61 for examinations with or without wheeze. While overall intralistener agreement was substantial (PABAK 0.62–0.68), reflecting the reproducibility of examination interpretations, there was modest variability from panellist to panellist, irrespective of the examination result analysed. The panel’s overall between listener PABAKs for control interpretations were in the moderate to substantial range (0.40–0.65), depending on the lung sound model (see online supplementary table 1).

10.1136/bmjresp-2017-000193.supp1 Supplementary Table 1

**Table 2:** Digital lung sound examination agreement between and within primary listeners in cases, stratified by listener*

Between listener agreement			Listener (N=number of recordings interpreted by each panellist)							
Dichotomous lung examination group		Overall (n=709)	Listener #1 (n=183)	Listener #2 (n=172)	Listener #3 (n=182)	Listener #4 (n=159)	Listener #5 (n=183)	Listener #6 (n=187)	Listener #7 (n=175)	Listener #8 (n=177)
Abnormal or normal†	Agreement, n (%)	531 (74.9)	143 (78.2)	124 (72.0)	135 (74.2)	115 (72.4)	128 (70.0)	148 (79.2)	123 (70.2)	146 (82.4)
	Kappa statistic (95% CI)	0.45 (0.40 to 0.50)	0.49	0.41	0.44	0.43	0.37	0.52	0.31	0.60
	PABAK (95% CI)‡	0.50 (0.45 to 0.54)	0.56	0.44	0.48	0.45	0.40	0.58	0.41	0.65
Crackle or no crackle	Agreement, n/N (%)	500/709 (70.6)	114/183 (62.2)	121/172 (70.4)	126/182 (69.2)	105/159 (66.0)	134/183 (73.2)	132/187 (70.6)	131/175 (74.8)	137/177 (77.4)
	Kappa statistic (95% CI)	0.40 (0.35 to 0.44)	0.30	0.32	0.36	0.29	0.44	0.40	0.50	0.54
	PABAK (95% CI)§	0.41 (0.36 to 0.46)	0.25	0.41	0.39	0.32	0.46	0.41	0.50	0.55
Wheeze or no wheeze	Agreement, n/N (%)	515 (72.6)	141/183 (77.0)	120/172 (69.8)	131/182 (72.0)	121/159 (76.2)	120/183 (65.6)	136/187 (72.8)	119/175 (68.0)	142/177 (80.2)
	Kappa statistic (95% CI)	0.45 (0.41 to 0.50)	0.54	0.40	0.44	0.52	0.30	0.45	0.38	0.60
	PABAK (95% CI)¶	0.45 (0.41 to 0.50)	0.54	0.40	0.44	0.52	0.31	0.46	0.36	0.61

**Table:** 

Within listener agreement			Listener (N=number of recordings interpreted by each panellist)							
Dichotomous lung examination group		Overall (n=136)	Listener #1 (n=18)	Listener #2 (n=13)	Listener #3 (n=20)	Listener #4 (n=19)	Listener #5 (n=18)	Listener #6 (n=17)	Listener #7 (n=16)	Listener #8 (n=15)
Abnormal or normal†	Agreement, n (%)	110 (80.8)	16 (88.8)	11 (84.6)	14 (70.0)	14 (73.6)	16 (88.8)	16 (94.2)	11 (68.8)	12 (80.0)
	Kappa statistic (95% CI)	0.61 (0.48 to 0.75)	0.75	0.68	0.42	0.44	0.73	0.77	0.23	0.55
	PABAK (95% CI)**	0.62 (0.49 to 0.75)	0.78	0.69	0.40	0.47	0.78	0.88	0.38	0.60
Crackle or no crackle	Agreement, n/N (%)	112 (82.4)	15 (83.4)	13 (100.0)	12 (60.0)	17 (89.4)	17 (94.4)	15 (88.2)	12 (75.0)	11 (73.4)
	Kappa statistic (95% CI)	0.62 (0.48 to 0.76)	0.67	1.00	0.20	0.46	0.82	0.77	0.50	0.47
	PABAK (95% CI)††	0.65 (0.52 to 0.78)	0.67	1.00	0.2	0.79	0.89	0.77	0.5	0.47
Wheeze or no wheeze	Agreement, n/N (%)	114 (83.8)	14 (77.8)	11 (84.6)	16 (80.0)	16 (84.2	17 (94.4)	16 (94.2)	13 (81.2)	11 (73.4)
	Kappa statistic (95% CI)	0.67 (0.55 to 0.80)	0.56	0.68	0.57	0.62	0.77	0.85	0.63	0.47
	PABAK (95% CI)‡‡	0.68 (0.55 to 0.80)	0.56	0.69	0.60	0.68	0.89	0.88	0.63	0.47

*Excludes uninterpretable primary listener lung examination results for 83/792 cases. Primary listeners were the first two listeners randomly assigned to interpret a lung sound examination.

†Crackle and/or wheeze (abnormal) or no crackle and/or wheeze (normal).

‡Prevalence index 0.30, bias index −0.04.

§Prevalence index −0.15, bias index 0.01.

¶Prevalence index 0.1, bias index −0.05.

**Prevalence index 0.13, bias index 0.03.

††Prevalence index −0.28, bias index 0.

‡‡Prevalence index −0.13, bias index 0.03.

PABAK, prevalence-adjusted, bias-adjusted kappa statistic.

We also sought to identify predictors of primary listener disagreement in 987 cases and controls, after omitting 83 cases and 23 controls with uninterpretable examinations by at least one primary listener (table 3). We found predictors of disagreement for identifying examinations with or without either crackles and/or wheeze to be case status compared with controls (adjusted OR (aOR) 0.65 (95% CI 0.47 to 0.90)), ≥3 uninterpretable chest positions (aOR 2.05 (1.51 to 2.78)), intermittent crying (aOR 1.56 (1.16 to 2.11)) and upper airway noises (aOR 2.23 (1.62 to 3.07)). Upper airway noises were also a predictor of disagreement for determining whether an examination had crackles or not (aOR 2.15 (1.55 to 2.99)) or did or did not have wheezing (aOR 2.95 (2.12 to 4.11)) (see online supplementary table 2). Tachypnoea (aOR 1.76 (1.15 to 2.67)) and PERCH sites of Bangladesh (OR 1.91 (1.21 to 3.04)), Kenya (OR 1.87 (1.13 to 3.10)) and The Gambia (OR 2.72 (1.67 to 4.42)) predicted disagreement for recognising or not recognising crackles, while intermittent crying (aOR 1.62 (1.20 to 2.18)) predicted disagreement for identifying whether an examination did or did not have wheeze (see online supplementary table 2).

10.1136/bmjresp-2017-000193.supp2 Supplementary Table 2

**Table 3:** Factors associated with primary listener disagreement for cases and controls, stratified by lung examination result*

Dichotomous lung examination group	Characteristic	Disagreement, n/N (%)	OR (95% CI)	p Value	Adjusted OR (95% CI)†	p Value
Abnormal or normal‡	All cases and controls (n=987)	262 (26.6)	–	–	–	–
	Cases only (n=709)	178 (25.1)	0.77 (0.57 to 1.05)	0.10	0.65 (0.47 to 0.90)	<0.01
	Controls only (n=278)	84 (30.2)	–	–	–	–
	Age 1–11 months (n=601)	163 (27.1)	1.08 (0.81 to 1.44)	0.61	–	–
	Age 12–59 months (n=386)	99 (25.7)	–	–	–	–
	Tachypnoea§ (n=592)	152 (25.7)	0.92 (0.68 to 1.22)	0.55	–	–
	No tachypnoea (n=383)	105 (27.4)	–	–	–	–
	>3 uninterpretable chest positions (n=344)	121 (35.2)	1.93 (1.45 to 2.58)	<0.001	2.05 (1.51 to 2.78)	<0.001
	<3 uninterpretable chest positions (n=643)	141 (21.9)	–	–	–	–
	Intermittent crying (n=514)	161 (31.3)	1.68 (1.26 to 2.24)	<0.001	1.56 (1.16 to 2.11)	<0.01
	No intermittent crying (473)	101 (21.4)	–	–	–	–
	Upper airway noises (n=607)	189 (31.1)	1.90 (1.40 to 2.59)	<0.001	2.23 (1.62 to 3.07)	<0.001
	No upper airway noises (n=380)	73 (19.2)	–	–	–	–
	PERCH site	–	–	0.27	–	–

*Excludes uninterpretable primary listener lung examination results in both cases and controls. Eighty-three out of 792 cases and 23/301 controls were excluded. Primary listeners were the first two listeners randomly assigned to interpret a lung sound examination.

†Adjusted for all characteristics associated with disagreement at the significance level of 0.20.

‡Crackle and/or wheeze (abnormal) or no crackle and/or wheeze (normal).

§Tachypnoea defined as follows: <2 months: >60 breaths/minute; 2–11 months: >50 breaths/minute; 12–59 months: >40 breaths/minute.

PERCH, Pneumonia Etiology Research for Child Health.

Table 4 reports the final listening panel’s lung examination results for all 1093 PERCH participants, stratified by case–control status and study site. Please see the online supplementary material for lung sound examples of normal, crackle and wheeze. After full adjudication, the panel classified 5.6% (17/301) of all controls and 6.3% (50/792) of cases as uninterpretable (p=0.67). Among all interpretable lung examinations in controls, the proportion with an abnormal examination (defined as any crackle or any wheeze) did not differ by those with and without respiratory illnesses (17.0% (16/94) acute respiratory illness (ARI) versus 14.2% (27/190) non-ARI; p=0.54). Of non-ARI controls, 8.4% (16/190) had wheezing, 2.6% (5/190) crackles and 3.2% (6/190) both. A substantially higher proportion of cases (62.0% (460/742)), than all controls (15.1% (43/284)), had an abnormal lung examination (p<0.001). Wheezing was the most prevalent abnormal examination, heard in 49.9% of cases (370/742) and 12.7% of all controls (36/284). While crackles were commonly heard among cases (39.1% (290/742)), panellists heard them more frequently with wheezing than in isolation (27.0% (200/742) vs 12.1% (90/742), p<0.001). Panellists identified crackles in 6.0% of controls (17/284).

**Table 4:** Final full listening panel classification of all digital lung sound examinations in PERCH participants stratified by case–control status and study site

	Total*	Normal n (%)	Abnormal n (%)†	Crackle only n (%)	Wheeze only n (%)	Crackle and wheeze n (%)
Controls						
Total*	284	241 (84.9)	43 (15.1)‡	7 (2.5)	26 (9.2)	10 (3.5)
Kenya	3	3 (100.0)	0 (0.0)	0 (0.0)	0 (0.0)	0 (0.0)
The Gambia	46	38 (82.6)	8 (17.4)	1 (2.2)	4 (8.7)	3 (6.5)
Zambia	36	26 (72.2)	10 (27.8)	0 (0.0)	9 (25.0)	1 (2.8)
South Africa	9	7 (77.8)	2 (22.2)	0 (0.0)	0 (0.0)	2 (22.2)
Bangladesh	23	15 (65.2)	8 (34.8)	3 (13.0)	5 (21.7)	0 (0.0)
Thailand	167	152 (91.0)	15 (9.0)	3 (1.8)	8 (4.8)	4 (2.4)

**Table:** 

Cases						
Total*	742	282 (38.0)	460 (62.0)‡	90 (12.1)§	170 (22.9)	200 (27.0)§
Kenya	125	62 (49.6)	63 (50.4)	14 (11.2)	27 (21.6)	22 (17.6)
The Gambia	80	8 (10.0)	72 (90.0)	6 (7.5)	40 (50.0)	26 (32.5)
Zambia	234	117 (50.0)	117 (50.0)	34 (14.5)	40 (17.1)	43 (18.4)
South Africa	94	38 (40.4)	56 (59.6)	15 (16.0)	12 (12.8)	29 (30.9)
Bangladesh	145	34 (23.4)	111 (76.6)	13 (9.0)	30 (20.7)	68 (46.9)
Thailand	64	23 (35.9)	41 (64.1)	8 (12.5)	21 (32.8)	12 (18.8)

*Seventeen out of 301 controls (5.6%) and 50/792 cases (6.3%) were excluded since the final panel lung examination result was uninterpretable; p value=0.666. Note that the denominators reflect the final full panel result (excluding uninterpretable results) *including* interpretations by the third and final two listeners, as necessary, who adjudicated any discordant results between primary listeners or between the first three listeners, respectively.

†Any crackle or any wheeze.

‡Proportion of abnormal lung sound examinations in cases (62.0%) versus all controls (15.1%); p value<0.001.

§Proportion of lung examinations with crackle only (12.1%) versus crackle and wheeze (27.0%); p value<0.001.

ARI, acute respiratory infection; PERCH, Pneumonia Etiology for Child Health.

## Discussion

We leveraged the largest paediatric pneumonia aetiology study in nearly 30 years to collect digitally recorded lung sounds from 1093 children with and without pneumonia in six high pneumonia burden African and Asian countries. Our data demonstrate that recording quality digital lung sounds from children in a range of noisy, crowded clinical settings in low-resource countries is feasible. This study also showed that with a panel of standardised paediatric experts, adjudication procedures modelled after the WHO chest radiograph process,^25^ and lung sound definitions pragmatically adapted from American Thoracic Society guidelines,^17 23^paediatric digital lung sound examinations can be interpreted acoustically by humans with moderate reliability; case–control comparisons suggest this methodology is valid.

The reliability achieved by this study’s expert listening panel compares favourably to paediatric literature from resource-rich settings. Two small studies from the USA and UK examined agreement levels between paediatricians acoustically identifying abnormal lung examinations in children with standard stethoscopes. These authors reported kappas of 0.18–0.70 for wheeze, 0.46 for crackle and 0.30 for all abnormal sounds.^8 26^ Our panel achieved moderate agreement for these lung sound categories (kappas of 0.40–0.45, PABAKs of 0.41–0.50). In addition to using a traditional stethoscope, these studies differed from ours by using unstandardised listeners who were not blinded to patients and had smaller sample sizes with wider CIs. A recent study of 120 German infants included digital auscultation and examined agreement levels for expiratory wheezing between three blinded physicians.^27^ The authors reported moderate agreement (Fliess’ kappa 0.54 (95% CI 0.52 to 0.57)) from recordings collected in a quiet clinical setting. Our listener agreement for wheezing was also moderate (kappa 0.45, PABAK 0.45), despite recording in noisy clinical environments. After comparison with the published literature, our results suggest that this study’s methodology, which includes the application of a novel software program that filters ambient noises from lung recordings,^22^ and interpretation procedures may improve the overall consistency of between listener reliability, compared with traditional auscultation, and may perform with comparable reliability to lung sounds recorded by digital stethoscopes in quieter environments.

Our study had several unanticipated results. Our panellists found a surprisingly high proportion of controls without ARI (14.2%) to have abnormal digital lung sound examinations. Two factors may explain these findings. First, lung sound recordings from sensitive digital stethoscopes may capture more subclinical abnormalities in healthy subjects compared with acoustic stethoscopes. Subclinical wheezing and crackles, especially in asymptomatic children in developing countries, may reflect ongoing small airway inflammation triggered by asthma or poor air quality,^28^ for example, or pre-existing lung damage or resolving inflammation from a prior illness like pneumonia.^29^ Non-pathological crackles are also possible and have been reported to frequently occur during inspiration in healthy adults after deeply exhaling to the lung’s residual volume.^30^ Interestingly, the authors of a recent systematic review also found wheezing in 1%–5% and crackles in 7%–37% of healthy, asymptomatic adults from studies in the USA, but noted a gap in paediatric data, as there were no published studies that included healthy children.^31^ Second, our expert listeners were blinded to the case–control status and also the visual cues that exist during a live patient encounter, and this may have increased false positive examinations; by looking at and listening to the patient at the same time, visual cues can help a clinician distinguish between wheezes, upper airway sounds such as cries, normal vocalisations, or transmitted nasal congestion (ie, the clinician can see the child crying, vocalising or rhinorrhea), or crackles and movement artefact (ie, the clinician can see the child moving), all of which can overlap in their amplitude and frequency profiles.^32^ Notably, the majority of control lung sounds were recorded from Thailand (58.8% (167/284)), as this was the only site to enrol all controls into the digital auscultation substudy. While it is possible that the over-representation of controls from Thailand may have further reduced the proportion of abnormal sounds heard among controls (91.0% of lung recordings from Thailand controls were normal), Thailand controls, since they were enrolled consecutively, were less susceptible to selection bias than other PERCH sites. For this reason, we feel the Thailand controls, and therefore the overall control population in this dataset, are more likely to be representative of the true control population.

While PERCH cases all met severe or very severe WHO clinical pneumonia criteria, 38.0% had normal digital lung sound examinations, suggesting an absence of lower respiratory disease. By design, the WHO algorithm for the management of severe and very severe pneumonia is non-specific for pneumonia and as expected we found that a significant fraction of the children meeting these definitions do not have auscultatory findings of lower respiratory tract disease.^4–7^ This observation is further reflected by over a third of all PERCH cases having a normal chest radiograph.^33^ However, selection bias may also have influenced lung sound findings; The Gambia and South Africa sampled cases by convenience, and Thailand, Bangladesh, The Gambia and South Africa enrolled cases into the digital auscultation substudy for less than 12 months, increasing the likelihood of seasonal variation in their data. Our data did reveal, as expected, that cases had a markedly higher proportion of abnormal lung examinations compared with all controls. Wheezing was decidedly prevalent among cases, regardless of PERCH site, which may imply that small airway inflammation due to viruses and asthma are common in WHO pneumonia. While crackles were also common, they were more frequently heard with wheezes than alone, a pattern characteristic of viral bronchiolitis.^34^ Importantly, while this study was not designed to assess the validity of digital lung examinations compared with a reference standard, our finding that more than twice as many controls, compared with cases, had normal lung sounds suggests that the methodology we developed is valid. Forthcoming analyses will explore the associations between digital lung examinations and etiological and radiographic disease endpoints to provide a better understanding of how digital auscultation may perform as a respiratory diagnostic in low-resource settings. Additionally, we plan to analyse how interpretations of digital lung recordings compare with interpretations of lung sounds using traditional acoustic stethoscopes. If we find interpretations between these two listening modalities to be comparable, this would support lung recordings as a possible educational tool for healthcare providers who have limited training in lung auscultation but use traditional acoustic stethoscopes to examine children.

Our multivariate models suggest areas of procedural or device limitations that are specific to children, and if strengthened, could improve paediatric lung sound recording quality and listener agreement. For instance, our data suggest that innovations to filter out upper airway noises like vocalisations or nasal secretions are needed. Devices or procedures that are more ‘child friendly’ may also help practitioners with less paediatric experience, soothe the child and collect sounds not contaminated by crying or movements. While not identified as a predictor of disagreement, panellists requested cues to help listeners better identify the respiratory phase as either inspiration or expiration. Other studies have used respiratory belts for this purpose, and this could be explored for feasibility and effectiveness in low-resource settings.^27^

In conclusion, this multicountry study provides evidence that quality lung sounds can be recorded from children in noisy clinical environments and interpreted by paediatric experts with moderate reliability. With further research and refinement of digital auscultation technology, recorded lung sounds may eventually play a greater role in the identification, categorisation and management of children with respiratory illness in developing countries.

10.1136/bmjresp-2017-000193.supp3 Supplementary material 3

10.1136/bmjresp-2017-000193.supp4 Supplementary material 4

10.1136/bmjresp-2017-000193.supp5 Supplementary material 5

## References

[1] Liu L, Oza S, Hogan D, et al Global, regional, and national causes of child mortality in 2000-13, with projections to inform post-2015 priorities: an updated systematic analysis. Lancet 2015;385:430–40.doi:10.1016/S0140-6736(14)61698-6 25280870 10.1016/S0140-6736(14)61698-6

[2] World Health Organization. IMCI: the integrated approach. Geneva, 1997.

[3] Sazawal S, Black RE Pneumonia Case Management Trials Group. Effect of pneumonia case management on mortality in neonates, infants, and preschool children: a meta-analysis of community-based trials. Lancet Infect Dis 2003;3:547–56.doi:10.1016/S1473-3099(03)00737-0 12954560 10.1016/s1473-3099(03)00737-0

[4] Cardoso MR, Nascimento-Carvalho CM, Ferrero F, et al Adding fever to WHO criteria for diagnosing pneumonia enhances the ability to identify pneumonia cases among wheezing children. Arch Dis Child 2011;96:58–61.doi:10.1136/adc.2010.189894 20870628 10.1136/adc.2010.189894

[5] Hazir T, Nisar YB, Qazi SA, et al Chest radiography in children aged 2-59 months diagnosed with non-severe pneumonia as defined by World Health Organization: descriptive multicentre study in Pakistan. BMJ 2006;333:629 doi:10.1136/bmj.38915.673322.80 16923771 10.1136/bmj.38915.673322.80 PMC1570841

[6] Hazir T, Nisar YB, Abbasi S, et al Comparison of oral amoxicillin with placebo for the treatment of world health organization-defined nonsevere pneumonia in children aged 2-59 months: a multicenter, double-blind, randomized, placebo-controlled trial in Pakistan. Clin Infect Dis 2011;52:293–300.doi:10.1093/cid/ciq142 21189270 10.1093/cid/ciq142

[7] Puumalainen T, Quiambao B, Abucejo-Ladesma E, et al Clinical case review: a method to improve identification of true clinical and radiographic pneumonia in children meeting the World Health Organization definition for pneumonia. BMC Infect Dis 2008;8:95 doi:10.1186/1471-2334-8-95 18644109 10.1186/1471-2334-8-95 PMC2492864

[8] Margolis P, Gadomski A The rational clinical examination. does this infant have pneumonia? JAMA 1998;279:308–13. 9450716 10.1001/jama.279.4.308

[9] Vyshedskiy A, Alhashem RM, Paciej R, et al Mechanism of inspiratory and expiratory crackles. Chest 2009;135:156–64.doi:10.1378/chest.07-1562 18689587 10.1378/chest.07-1562

[10] Metlay JP, Kapoor WN, Fine MJ Does this patient have community-acquired pneumonia? diagnosing pneumonia by history and physical examination. JAMA 1997;278:1440–5. 9356004

[11] McCarthy PL, Lembo RM, Baron MA, et al Predictive value of abnormal physical examination findings in ill-appearing and well-appearing febrile children. Pediatrics 1985;76:167–71. 4022688

[12] Wipf JE, Lipsky BA, Hirschmann JV, et al Diagnosing pneumonia by physical examination: relevant or relic? Arch Intern Med 1999;159:1082–7. 10335685 10.1001/archinte.159.10.1082

[13] Gjłrup T, Bugge PM, Jensen AM Interobserver variation in assessment of respiratory signs. Physicians' guesses as to interobserver variation. Acta Med Scand 1984;216:61–6. 6485882 10.1111/j.0954-6820.1984.tb03772.x

[14] Ceresa CC, Johnston ID Auscultation in the diagnosis of respiratory disease in the 21st century. Postgrad Med J 2008;84:393–4.doi:10.1136/pgmj.2008.070474 18832398 10.1136/pgmj.2008.070474

[15] Murphy R Computerized multichannel lung sound analysis. Development of acoustic instruments for diagnosis and management of medical conditions. IEEE Eng Med Biol Mag 2007;26:16–19.doi:10.1109/MEMB.2007.289117 17278768 10.1109/memb.2007.289117

[16] Grenier MC, Gagnon K, Genest J, et al Clinical comparison of acoustic and electronic stethoscopes and design of a new electronic stethoscope. Am J Cardiol 1998;81:653–6.doi:10.1016/S0002-9149(97)00977-6 9514471 10.1016/s0002-9149(97)00977-6

[17] Murphy RL In defense of the stethoscope. Respir Care 2008;53:355–69. 18291053

[18] Gurung A, Scrafford CG, Tielsch JM, et al Computerized lung sound analysis as diagnostic aid for the detection of abnormal lung sounds: a systematic review and meta-analysis. Respir Med 2011;105:1396–403.doi:10.1016/j.rmed.2011.05.007 21676606 10.1016/j.rmed.2011.05.007 PMC3227538

[19] Levine OS, O'Brien KL, Deloria-Knoll M, et al The Pneumonia Etiology Research for Child Health Project: a 21st century childhood pneumonia etiology study. Clin Infect Dis 2012;54(Suppl 2):S93–S101.doi:10.1093/cid/cir1052 22403238 10.1093/cid/cir1052 PMC3297546

[20] Scott JA, Wonodi C, Moïsi JC, et al The definition of pneumonia, the assessment of severity, and clinical standardization in the Pneumonia Etiology Research for Child Health study. Clin Infect Dis 2012;54(Suppl 2):S109–S116.doi:10.1093/cid/cir1065 22403224 10.1093/cid/cir1065 PMC3297550

[21] Deloria-Knoll M, Feikin DR, Scott JA, et al Identification and selection of cases and controls in the Pneumonia Etiology Research for Child Health project. Clin Infect Dis 2012;54(Suppl 2):S117–S123.doi:10.1093/cid/cir1066 22403225 10.1093/cid/cir1066 PMC3297551

[22] Emmanouilidou D, McCollum ED, Park DE, et al Adaptive noise suppression of pediatric lung auscultations with real applications to Noisy clinical settings in developing countries. IEEE Trans Biomed Eng 2015;62:2279–88.doi:10.1109/TBME.2015.2422698 25879837 10.1109/TBME.2015.2422698 PMC4568755

[23] Pulmonary terms and symbols. A report of the ACCP-STS Joint Committee on Pulmonary Nomenclature. Chest 1975;67:583–93. 1126197

[24] Sim J, Wright CC The kappa statistic in reliability studies: use, interpretation, and sample size requirements. Phys Ther 2005;85:257–68. 15733050

[25] Cherian T, Mulholland EK, Carlin JB, et al Standardized interpretation of paediatric chest radiographs for the diagnosis of pneumonia in epidemiological studies. Bull World Health Organ 2005;83:353–9.doi:/S0042-96862005000500011 15976876 PMC2626240

[26] Elphick HE, Lancaster GA, Solis A, et al Validity and reliability of acoustic analysis of respiratory sounds in infants. Arch Dis Child 2004;89:1059–63.doi:10.1136/adc.2003.046458 15499065 10.1136/adc.2003.046458 PMC1719716

[27] Puder LC, Fischer HS, Wilitzki S, et al Validation of computerized wheeze detection in young infants during the first months of life. BMC Pediatr 2014;14:257 doi:10.1186/1471-2431-14-257 25296955 10.1186/1471-2431-14-257 PMC4287542

[28] Gordon SB, Bruce NG, Grigg J, et al Respiratory risks from household air pollution in low and middle income countries. Lancet Respir Med 2014;2:823–60.doi:10.1016/S2213-2600(14)70168-7 25193349 10.1016/S2213-2600(14)70168-7 PMC5068561

[29] Brower KS, Del Vecchio MT, Aronoff SC The etiologies of non-CF bronchiectasis in childhood: a systematic review of 989 subjects. BMC Pediatr 2014;14:4 doi:10.1186/s12887-014-0299-y 10.1186/s12887-014-0299-y PMC4275950 25492164

[30] Workum P, Holford SK, Delbono EA, et al The prevalence and character of crackles (rales) in young women without significant lung disease. Am Rev Respir Dis 1982;126:921–3.doi:10.1164/arrd.1982.126.5.921 7149458 10.1164/arrd.1982.126.5.921

[31] Oliveira A, Marques A Respiratory sounds in healthy people: a systematic review. Respir Med 2014;108:550–70.doi:10.1016/j.rmed.2014.01.004 24491278 10.1016/j.rmed.2014.01.004

[32] Workum P, DelBono EA, Holford SK, et al Observer agreement, chest auscultation, and crackles in asbestos-exposed workers. Chest 1986;89:27–9.doi:10.1378/chest.89.1.27 3940785 10.1378/chest.89.1.27

[33] Fancourt N, Deloria Knoll M, Baggett HC, et al Chest radiograph findings in childhood pneumonia cases from the multisite PERCH study. Clin Infect Dis. In Press 2017;64:S262–S270.doi:10.1093/cid/cix089 28575361 10.1093/cid/cix089 PMC5447837

[34] Smyth RL, Openshaw PJ Bronchiolitis. Lancet 2006;368:312–22.doi:10.1016/S0140-6736(06)69077-6 16860701 10.1016/S0140-6736(06)69077-6

