# Supplementary material for: Listening panel agreement and characteristics of lung sounds digitally recorded from children aged 1–59 months enrolled in the Pneumonia Etiology Research for Child Health (PERCH) case–control study
Source: BMJ Open Respir Res. 2017 Jun 30;4(1):e000193. doi: 10.1136/bmjresp-2017-000193 (PMC5531306; doi:10.1136/bmjresp-2017-000193)
Supplement: Supplementary Table 1 [file bmjresp-2017-000193supp001.pdf]

Supplementary Table 1. Digital lung sound examination agreement **between** primary listeners in ARI and non-ARI **controls**, stratified by listener[1]

Between listener agreement			Listener (N = number of recordings interpreted by each panelist)							
**Dichotomous lung examination group**		**Overall (N=278)**	**Listener #1 (N=73)**	**Listener #2 (N=67)**	**Listener #3 (N=68)**	**Listener #4 (N=74)**	**Listener #5 (N=63)**	**Listener #6 (N=61)**	**Listener #7 (N=78)**	**Listener #8 (N=72)**
**Abnormal or normal[2]**	**Agreement, n (%)**	194 (69.8)	44 (60.2)	52 (77.6)	50 (73.6)	56 (75.6)	46 (73.0)	47 (77.0)	40 (51.2)	53 (73.6)
	**Kappa statistic (95% CI)**	0.21 (0.13, 0.30)	0.13	0.27	0.35	0.29	0.03	0.35	0.06	0.22
	**PABAK (95% CI)[3]**	0.40 (0.32, 0.47)	0.21	0.55	0.47	0.51	0.46	0.54	0.03	0.47
**Crackle or no crackle**	**Agreement, n/N (%)**	229 (82.4)	50 (68.4)	61 (91.0)	57 (83.8)	65 (87.8)	55 (87.4)	51 (83.6)	60 (77.0)	59 (82.0)
	**Kappa statistic (95% CI)**	0.21 (0.11, 0.32)	0.16	0.21	0.26	0.34	-0.03	0.09	0.26	0.21
	**PABAK (95% CI)[4]**	0.65 (0.58, 0.71)	0.37	0.82	0.68	0.76	0.67	0.67	0.54	0.64
**Wheeze or no wheeze**	**Agreement, n/N (%)**	205 (73.8)	55 (75.4)	54 (80.6)	50 (73.6)	57 (77.0)	51 (81.0)	50 (82.0)	36 (46.2)	57 (79.2)
	**Kappa statistic (95% CI)**	0.14 (0.05-0.23)	0.16	0.32	0.21	0.19	0.14	0.37	-0.10	0.00
	**PABAK (95% CI)[5]**	0.48 (0.40, 0.55)	0.51	0.61	0.47	0.54	0.62	0.64	-0.08	0.58

ARI indicates acute respiratory infection; CI, confidence interval; PABAK, prevalence adjusted, bias adjusted kappa statistic.

[1]Excludes 23/301 uninterpretable primary listener lung examination results. Primary listeners were the first two listeners randomly assigned to interpret a lung sound examination.

[2]Crackle and/or wheeze (abnormal) or no crackle and/or wheeze (normal)

[3]Prevalence index -0.48, Bias index -0.03

[4]Prevalence index -0.75, Bias index 0.05

[5]Prevalence index -0.63, Bias index -0.08
