# Supplementary material for: Listening panel agreement and characteristics of lung sounds digitally recorded from children aged 1–59 months enrolled in the Pneumonia Etiology Research for Child Health (PERCH) case–control study
Source: BMJ Open Respir Res. 2017 Jun 30;4(1):e000193. doi: 10.1136/bmjresp-2017-000193 (PMC5531306; doi:10.1136/bmjresp-2017-000193)
Supplement: Supplementary Table 2 [file bmjresp-2017-000193supp002.pdf]

Supplementary Table 2. Factors associated with primary listener disagreement for cases and controls, stratified by lung examination result[1]

Dichotomous lung examination group	Characteristic	Disagreement, n/N (%)	OR (95% CI)	p value	Adjusted[2] OR (95% CI)	p value
Crackle or no crackle	**All cases and controls (N=987)**	258 (26.1%)	--	--	--	--
	**Cases only (N=709)**	209 (29.5%)	1.95 (1.38, 2.77)	<0.001	1.37 (0.82, 2.30)	0.23
	**Controls only (N=278)**	49 (17.6%)	--	--	--	--
	**Age 1-11 months (N=601)**	154 (25.6%)	0.93 (0.70, 1.25)	0.65	--	--
	**Age 12-59 months (N=386)**	104 (26.9%)	--	--	--	--
	**Tachypnea[3] (N=592)**	186 (31.4%)	2.09 (1.52, 2.85)	<0.001	1.76 (1.15, 2.67)	<0.01
	**No tachypnea (N=383)**	69 (18.0%)	--	--	--	--
	**≥3 uninterpretable chest positions (N=344)**	91 (26.5%)	1.03 (0.76, 1.38)	0.87	--	--
	**<3 uninterpretable chest positions (N=643)**	167 (26.0%)	--	--	--	--
	**Intermittent crying (N=514)**	138 (26.9%)	1.08 (0.81, 1.44)	0.60	--	--
	**No intermittent crying (473)**	120 (25.4%)	--	--	--	--
	**Upper airway noises (N=607)**	192 (31.6%)	2.20 (1.61, 3.02)	<0.001	2.15 (1.55, 2.99)	<0.001
	**No upper airway noises**	66 (17.4%)	--	--	--	--

	**(N=380)**					
	**PERCH site**	--	--	<0.001	--	<0.001
	**Thailand (N=227)**	45 (19.8%)	1.00	--	--	--
	**Bangladesh (N=165)**	53 (32.1%)	1.91 (1.21, 3.04)	--	--	--
	**Kenya (N=120)**	38 (31.7%)	1.87 (1.13, 3.10)	--	--	--
	**The Gambia (N=122)**	49 (40.2%)	2.72 (1.67, 4.42)	--	--	--
	**Zambia (N=258)**	50 (19.4%)	0.97 (0.62, 1.52)	--	--	--
	**South Africa (N=95)**	23 (24.2%)	1.29 (0.73, 2.29)	--	--	--
**Dichotomous lung examination group**	**Characteristic**	**Disagreement, n/N (%)**	**OR (95% CI)**	**p value**	**Adjusted[2] OR (95% CI)**	**p value**
**Wheeze or no wheeze**	**All cases and controls (N=987)**	267 (27.1%)	--	--	--	--
	**Cases only (N=709)**	194 (27.4%)	1.06 (0.77, 1.45)	0.73	--	--
	**Controls only (N=278)**	73 (26.3%)	--	--	--	--
	**Age 1-11 months (N=601)**	169 (28.1%)	1.15 (0.86, 1.54)	0.35	--	--
	**Age 12-59 months (N=386)**	98 (25.4%)	--	--	--	--
	**Tachypnea[3] (N=592)**	161 (27.2%)	1.07 (0.80, 1.44)	0.64	--	--
	**No tachypnea (N=383)**	99 (25.9%)	--	--	--	--
	**$\geq$3 uninterpretable chest positions (N=344)**	101 (29.4%)	1.19 (0.89, 1.60)	0.23	--	--

	**<3 uninterpretable chest positions (N=643)**	166 (25.8%)	--	--	--	--
	**Intermittent crying (N=514)**	161 (31.3%)	1.58 (1.19, 2.10)	<0.01	1.62 (1.20, 2.18)	<0.01
	**No intermittent crying (473)**	106 (22.4%)	--	--	--	--
	**Upper airway noises (N=607)**	209 (34.4%)	2.92 (2.11, 4.04)	<0.001	2.95 (2.12, 4.11)	<0.001
	**No upper airway noises (N=380)**	58 (15.3%)	--	--	--	--
	**PERCH site**	--	--	0.10	--	0.21

OR indicates Odds Ratio; CI, confidence interval; PERCH, Pneumonia Etiology Research for Child Health.

[1]Excludes uninterpretable primary listener lung examination results in both cases and controls. 83/792 cases and 23/301 controls were excluded. Primary listeners were the first two listeners randomly assigned to interpret a lung sound examination.

[2]Adjusted for all characteristics associated with disagreement at the significance level of 0.20.

[3]Tachypnea defined as follows: <2 months: >60 breaths/minute; 2-11 months: >50 breaths/minute; 12-59 months: >40 breaths/minute
